# Untargeted metabolomics approach to discriminate mistletoe commercial products

**DOI:** 10.1038/s41598-021-93255-z

**Published:** 2021-07-09

**Authors:** Cécile Vanhaverbeke, David Touboul, Nicolas Elie, Martine Prévost, Cécile Meunier, Sylvie Michelland, Valérie Cunin, Ling Ma, David Vermijlen, Cédric Delporte, Stéphanie Pochet, Audrey Le Gouellec, Michel Sève, Pierre Van Antwerpen, Florence Souard

**Affiliations:** 1 Univ. Grenoble Alpes, CNRS, DPM, 38000 Grenoble, France; 2 Université Paris-Saclay, CNRS, Institut de Chimie des Substances Naturelles, UPR 2301, 91198 Gif-sur-Yvette, France; 3 Structure et Fonction des Membranes Biologiques, Université libre de Bruxelles (ULB), 1050 Brussels, Belgium; 4 CHU Grenoble Alpes, Service de Biochimie et Biologie moléculaire et Toxicologie Environnementale, 38000 Grenoble, France; 5 Univ. Grenoble Alpes, CHU Grenoble Alpes, Plateforme GExiM, 38000 Grenoble, France; 6 Univ. Grenoble Alpes, CNRS, Grenoble INP, CHU Grenoble Alpes, TIMC-IMAG, Grenoble, France; 7 Department of Pharmacotherapy and Pharmaceutics (DPP), Université libre de Bruxelles (ULB), 1050 Brussels, Belgium; 8 Institute for Medical Immunology, Université libre de Bruxelles, 6041 Gosselies, Belgium; 9 ULB Center for Research in Immunology (U-CRI), Université libre de Bruxelles (ULB), Brussels, Belgium; 10 RD3-Pharmacognosy, Bioanalysis and Drug Discovery and Analytical Platform of the Faculty of Pharmacy, Université libre de Bruxelles (ULB), 1050 Brussels, Belgium

**Keywords:** Metabolomics, Secondary metabolism

## Abstract

Mistletoe (*Viscum album* L.) is used in German-speaking European countries in the field of integrative oncology linking conventional and complementary medicine therapies to improve quality of life. Various companies sell extracts, fermented or not, for injection by subcutaneous or intra-tumoral route with a regulatory status of anthroposophic medicinal products (European Medicinal Agency (EMA) assessment status). These companies as well as anthroposophical physicians argue that complex matrices composed of many molecules in mixture are necessary for activity and that the host tree of the mistletoe parasitic plant is the main determining factor for this matrix composition. The critical point is that parenteral devices of European mistletoe extracts do not have a standard chemical composition regulated by EMA quality guidelines, because they are not drugs, regulatory speaking. However, the mechanism of mistletoe’s anticancer activity and its effectiveness in treating and supporting cancer patients are not fully understood. Because of this lack of transparency and knowledge regarding the matrix chemical composition, we undertook an untargeted metabolomics study of several mistletoe extracts to explore and compare their fingerprints by LC-(HR)MS(/MS) and ^1^H-NMR. Unexpectedly, we showed that the composition was primarily driven by the manufacturer/preparation method rather than the different host trees. This differential composition may cause differences in immunostimulating and anti-cancer activities of the different commercially available mistletoe extracts as illustrated by structure–activity relationships based on LC–MS/MS and ^1^H-NMR identifications completed by docking experiments. In conclusion, in order to move towards an evidence-based medicine use of mistletoe, it is a priority to bring rigor and quality, chemically speaking.

## Introduction

Natural products from plants have always been a rich source of compounds for drug discovery. However, their use has decreased in the past two decades, partially because of technical barriers to screen natural multicomponent products or even more complex plant-matrices in high-throughput assays against a medicinal target (a biological receptor/enzyme). Nevertheless, natural products stay an important source of lead compounds for new medicines, despite reduced interest from pharmaceutical companies.

Commercial natural products are present in complementary medicine, particularly beside cancer treatments. Indeed, based on a systematic North-American and European consensus approach, integrative oncology (IO) has recently been defined as a “…*patient-centered, evidence-informed field of cancer care that utilizes mind and body practices, natural products, and/or lifestyle modifications from different traditions alongside conventional cancer treatments. IO aims to optimize health, quality of life, and clinical outcomes across the cancer care continuum and to empower people to prevent cancer and become active participants before, during, and beyond cancer treatment…”*^1^.

Among natural products widely used in oncology, European mistletoe (*Viscum album* L.) is well positioned. It is a popular hemiparasitic, evergreen shrub occurring in Europe, northwest Africa, southwest and central Asia. This plant grows on various trees and, depending on the host, subspecies have been described: *Viscum album* L. ssp. *album* is seen on deciduous trees, *Viscum album* ssp. *abietis* (Wiesb.) Abrom. occurs on fir, whereas *Viscum album* ssp. *austriacum* (Wiesb.) Volim. grows mainly on pine^2^. Different families of metabolites have been previously described from the European mistletoe^3–5^.

In Europe, patients often turn to mistletoe extracts in IO in particular in German anthroposophical centers. Little is known about the manufacturing process of these products with the exception of the presence or absence of a fermentation process. In a general way, an herbal medicinal product is prevalent as complementary and alternative medicine (CAM) throughout Europe and this market steadily grew during the past decades, with Germany owning the largest European herbal medicine market^6^. Legal regulations have been developed since the late twentieth century to ensure quality, efficacy, and safety of herbal medicinal products (accessible mainly as “phyto-medicine”, herbal dietary supplements or homeopathic products). However, European mistletoe parenteral products have a special regulatory status as gold standard of anthroposophical medicine products (AMP). In German-speaking countries, mistletoe extracts are available as approved drugs (based on monographs of the C and E commissions of the German Federal Institute for Drugs and Medical Devices) use in anthroposophical medicine. It is practiced in integration with conventional medicine, in parallel of cancer chemotherapy^7,8^. Today, AMP are not specifically regulated by the EU (except in German-speaking countries). Anthroposophic products are produced (1) according to a homeopathic manufacturing method described in the European Pharmacopoeia or (2) in absence thereof, in German or Swiss pharmacopoeia. (3) They can also be produced according to a specific “anthroposophic” manufacturing method. In European pharmacopeia, mistletoe extracts are not present. In France, mistletoe extracts (Weleda) have been sold until 2018 through homeopathic legislation. Now in France, it is not possible to buy them in official pharmacy. They are not sold as a drug in the United States. To obtain these products, consumers simply go on internet. What consumers do not know is that manufacturers are not obliged to respect drug standards, particularly for the chemical composition, control and standardization. On one hand, the composition of a mistletoe extract sold by one manufacturer or another is not necessarily homogenous. On the other hand, the therapeutic indications (according to the manufacturer product information file) remain substantially unchanged from one manufacturer to another.

Another point bringing more complexity has to be considered. Three sellers in Europe provide various extracts indicating the host and the presence or the absence of fermentation. Considering the first point and being a parasitic plant, mistletoe has a chemical composition influenced by the host tree. What is interesting, is that some of the metabolites present in mistletoe extracts are not produced by the plant but obtained from the host tree. In particular, chemical composition of xylem sap and seasonal and spatial variation in carbohydrates or amino-compounds have been published^9,10^. For another example, the presence of specific alkaloids was noted in mistletoe growing on plants which synthesize these compounds^11^. As a matter of fact, the chemical composition of mistletoe is also dependent on the seasons. Considering the chemical composition various review articles have published a detailed report of chemical constituents of *Viscum album* L^4,12^. The most famous compounds are proteins as cysteine-rich highly basic proteins viscotoxins and mistletoe lectins. These proteins are often highlighted as the key molecules responsible for the bioactivity^13–16^. Polysaccharides, triterpenes, lupanes, phytosterols, fatty acids, flavonoids, phenylpropanoids and phenolic acids have been also described. According to authors, these secondary metabolites would be also necessary for optimal activity^3,13^. The seasonal dynamics of proteins of leaves show a pronounced peak of maximal viscotoxin concentrations in June, whereas mistletoes lectins accumulate to a maximum amount in December. June and December, as key moments in the physiological state and correspond to the harvesting seasons according to the information provided by the manufacturers^3^. Considering now the second point—the bacterial fermentation—it is difficult to find robust information in peer-review articles. Ribéreau-Gayon et al. in 1986 have described that fermented Iscador contained a low amount of lectins, approximately 100 ng/ml, while unfermented Iscador contained about 10 times more^17^.

Based on the hypothesis that mistletoe products are potentially different in terms of composition depending on the season, the host, the processes (depending on the presence or the absence of the fermentation and extraction method…), we decided to explore the chemical fingerprints of products of the 3 sellers in Europe thanks to metabolomics technologies. Indeed, with these technics, rapid identifications of novel compounds in complex mixtures of natural products is now facilitated, with the additional help of molecular networking, and provides a way to monitor the production of target molecules^18–20^. An untargeted metabolomics analysis was performed on 17 extracts of mistletoe commercial products using liquid-chromatography coupled to high-resolution mass spectrometry LC-HRMS and ^1^H-NMR. To completely rationalize the chemical composition, molecular networks have been constructed based on LC-HRMS/MS raw data. Finally, a peculiar attention was put on abundant component(s) in Abnobaviscum extract (host *pini*) that could participate to its recently described immunostimulating anti-cancer activity^21^.

## Results

We collected mistletoe injectable preparations of 3 manufactures. The leaders of the market are two German compagnies, Abnoba GmbH and Helixor, and a Swiss multinational, Weleda. They provided us with different products. Weleda resells the products of the Swiss company Iscador AG under two commercial names, Weleda (available on the French market until 2019) and Iscador. Hence, Weleda products have been considered as Iscador products replicates, as another batch. Those samples have been completed by Abnoba and Helixor commercial products (Table 1). There is a certain confidentiality around the industrial process, we only know that some products were fermented and others were not; the fermentation process and strains used for it being not clearly indicated (except for Iscador company that claimed a fermentation by *Lactobacilli*).

**Table 1:** Description of the commercial studied extracts.

Commercial names	Pharmaceutical forms: doses (in mg/mL) (studied form in bold)	Type of extract	Abbreviation used
AbnobaVISCUM: Aceris, Amygdali, Betulae, Crataegi, Fraxini, Mali, Pini, Quercus	Injectable solution: 0.02, 0.2, 2, **20**	**Unfermented** fresh juice	Aac, Aam, Ab, Ac, Af, Am, Ap, Aq
Helixor A (Abietis), Helixor M (Mali), Helixor P (Pini)	Injectable solution: 0.01, 0.1, 1, 5, 10, 20, 30, **50**	Aqueous **unfermented** extract from fresh juice herb (1:20)	Hab, Hm, Hp
Iscador A (Abietis), Iscador M (Mali), Iscador P (Pini)	Injectable solution: 0.0001, 0.001, 0.01, 0.1, 1, 10, **20**	Aqueous extract from fresh juice herb + **fermentation**	Iab, Im, Ip
Weleda M (Mali), Weleda P (Pini), Weleda Q (Quercus)	Injectable solution: 0.0001, 0.001, 0.01, 0.1, 1, 10, **20**	Aqueous extract from fresh juice herb + **fermentation**	Wm, Wp, Wq

For the abbreviation, the first capital letter corresponds to the company name and the lowercase letters indicate the host tree (ab: *abietis*, ac: *aceris*, am: *amygdali*, b: *betulae*, c: *crataegi*, f: *fraxini*, m: *mali*, p: *pini*, q: *quercus*).

### Untargeted metabolomics focusing on manufacturers and annotation using molecular networks

**Figure 1:**
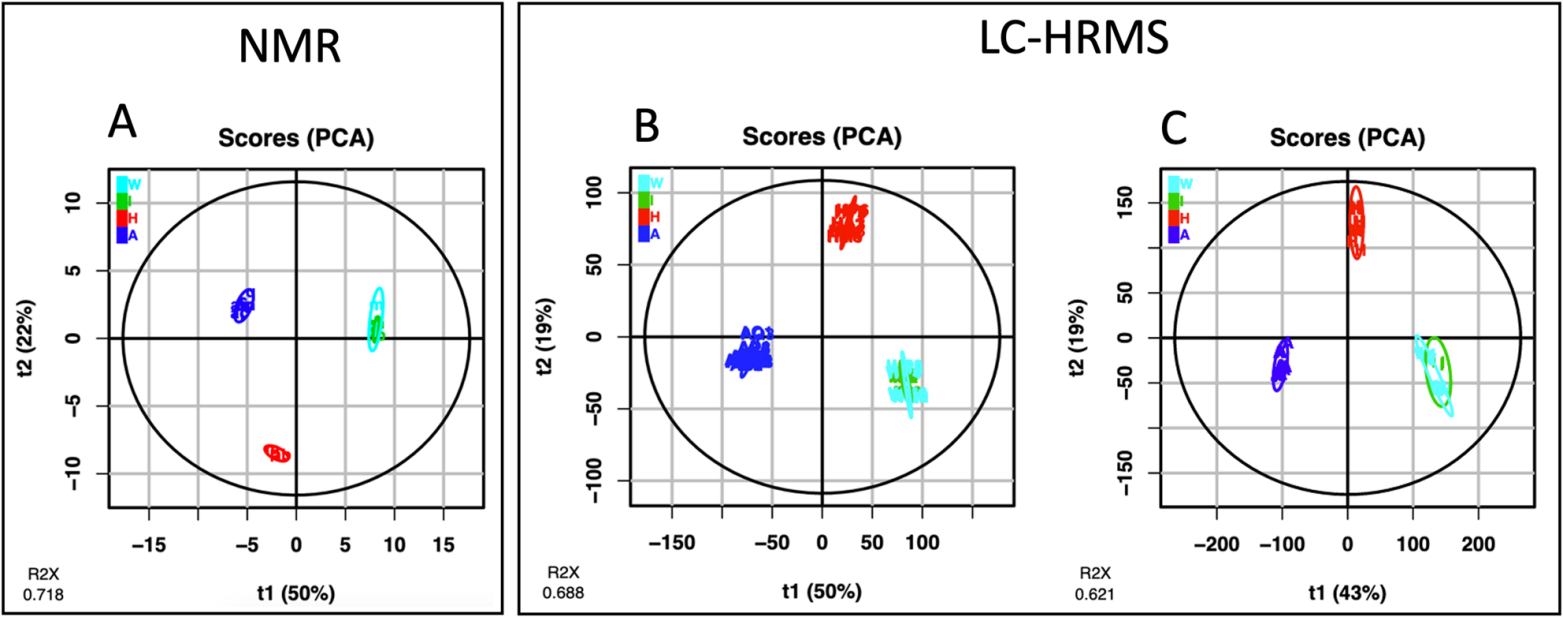
Principal Component Analysis (PCA) of *Viscum album* L. extracts: blue, Abnoba (A); red, Helixor (H); green, Iscador (I); cyan, Weleda (W). (**A**(: based on ^1^H-NMR spectra (resonance intervals of 0.02 ppm). (**B**) and (**C**): based on LC-HRMS data, post-processed with W4M online platform (XCMS) or MZmine, respectively. Explained variance of principal components is reported in brackets on the axis labels.

The metabolite profiles of the *Viscum album* L. extracts were analyzed by proton nuclear magnetic resonance (^1^H-NMR) and LC-HRMS, and have been proceeded and visualized using PCA (Fig. 1) and heatmaps (Supplementary Fig. S1) for a better visualization of the feature repartition. First, untargeted metabolomics analysis of 17 samples of *Viscum album* L. extracts, using ^1^H-NMR, yielded 304 features (buckets), which were statistically modeled using PCA. The PCA plot (after Log_10_ transformation and Pareto scaling) with the duplicates for 6 extracts was very similar to the one with the mean of the duplicates. Thus, we choose to present the mean of the duplicates (Fig. 1A). The two first components of the PCA model accounted for 72% of the variance set. The PCA plot clearly separated the samples into three groups corresponding exactly to the three producers: Abnoba, Helixor and Iscador and Weleda.

Untargeted metabolomic analysis of the *Viscum album* L. have been also proceeded by LC-HRMS. Two different data processing methods were currently used by metabolomics community for LC–MS datasets. The first one was XCMS, implemented in W4M free online platform^22^ and the second one, MZmine 2^23^, the open-source framework for processing, visualization and analysis of mass spectrometry based molecular profile data. It should be noted here that the use of MZmine for LC–MS/MS dataset is necessary to build molecular networks. To ensure that both data processing give similar results, we tested them on the same LC–MS dataset.

First of all, W4M was used^22^. This infrastructure is a user-friendly online platform for metabolomics data pre-processing and analysis. For the pre-processing, W4M platform uses XCMS tools. Seventeen samples yielded 8928 marker ions (unique retention time − *m/z* ion pairings) for 70 objects (17 commercial samples in triplicate/Quality controls/Blanks), which were statistically modeled using PCA (Fig. 1B). The replicates of each product were overlaid on the PCA plot (after Log_10_ transformation and Pareto scaling), indicating repeatability of LC–MS analysis. The 2 first-components of PCA model accounted for 69% of the variance in the sample set. One replicate of Ip sample has been considered as an outlier and has been taken off of the analysis. As by NMR, PCA plot clearly separated the samples into three groups corresponding exactly to the three producers. Secondly, MZmine treatment was used and for a better comparison, data processed by MZmine were visualized with the same tool than those processed by XCMS, i.e., W4M. The number of features was about twice larger with 19,476 marker ions than with W4M-XCMS pre-processing but the PCA obtained was very similar to the first one (Fig. 1B,C) which reinforces the validity of the MZmine processing for LC–MS/MS data. These observations suggest that the results obtained with MZmine was completely comparable to that of W4M-XCMS and that the former was richer. Thus, for the molecular network, we kept the MZmine processing.

**Figure 2:**
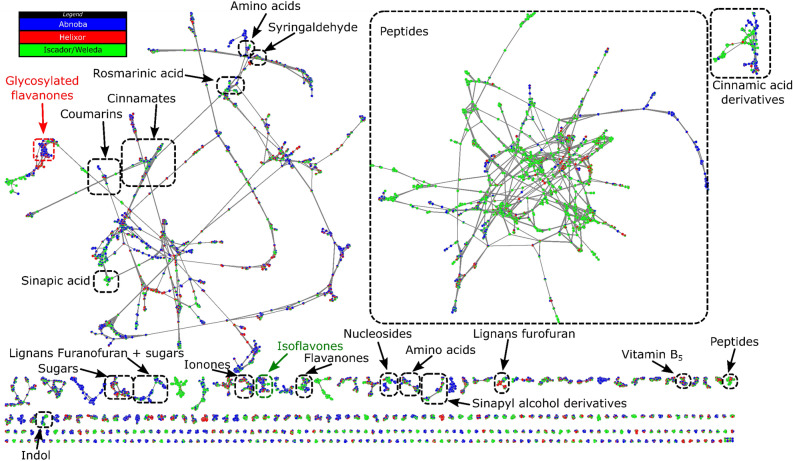
Molecular network of specialized metabolites annotated in crude extracts of *Viscum album* L. by LC-HRMS/MS analysis. Cluster annotation was performed using databases from MetGem. Pie charts inside nodes denotes abundance while colors correspond to the producers where the feature was found. The cluster related to isoflavones was circled in green and the one related to glycosylated flavanones in red.

Molecular network calculated by MetGem software^24^ of the crude extracts of *Viscum album* L. from the three producers enlightened 9306 nodes including 4042 isolated nodes (not shown in Fig. 2) and 5264 nodes grouped in clusters (Fig. 2). Each node corresponds to a unique couple of *m/z* of precursor ion and retention time values, linked to a MS/MS spectrum. Similarity between two MS/MS spectra was calculated based on fragments and neutral losses leading to a cosine score between 0 (no similarity) and 1 (complete similarity). Thus, nodes with a cosine score higher than 0.65 were connected to generate clusters. Annotations were based on interrogation of public databases (GNPS^25^, MS-Dial^26^ and ISDB^27^) from MetGem software leading to annotation at level 2^28^. The annotation is in good agreement with the previous report of metabolome of fermented aqueous extracts of *Viscum album* L.^29^, including a large amount of small peptides (second largest cluster in Fig. 2) together with data compiled with Urech and Baumgartner^7^ where various families of phenolic compounds were reported including derivatives of cinnamic, sinapinic, rosmarinic acids or flavones.

Pie charts enlightened different compositions of the extract depending on the producers (Fig. 2). For example, small peptides were abundant in Iscador/Weleda products but at low concentration in Helixor and Abnoba ones. On the contrary, glycosylated flavanones were more abundant in Abnoba and Helixor products but not detected in Iscador/Weleda ones. Therefore, molecular networks based on MS (relative quantification) and MS/MS (annotation) data allow distinguishing the composition of *Viscum album* L.^29^ from the three producers confirming the trends obtained by PCA analysis on MS features.

Using GNPS databases implemented in Metgem, we are able to identify 129 metabolites, with a cosine score over 0.90. As an example, we annotated in the flavanones cluster (Fig. 2) dihydroluteolin (cos score 0.98), isoxanthohumol (cos score 0.96) and homoeriodictyol more represented in Iscador/Weleda, and sakuranetin (cos score 0.95) more represented in Abnoba. In the Isoflavones cluster (Fig. 2, in green), we also annotated taxifolin (cos score 0.98) and dihydrokaempferol (cos score 0.94) more represented in Iscador/Weleda.

### Untargeted metabolomics and annotation focusing on host influence

**Figure 3:**
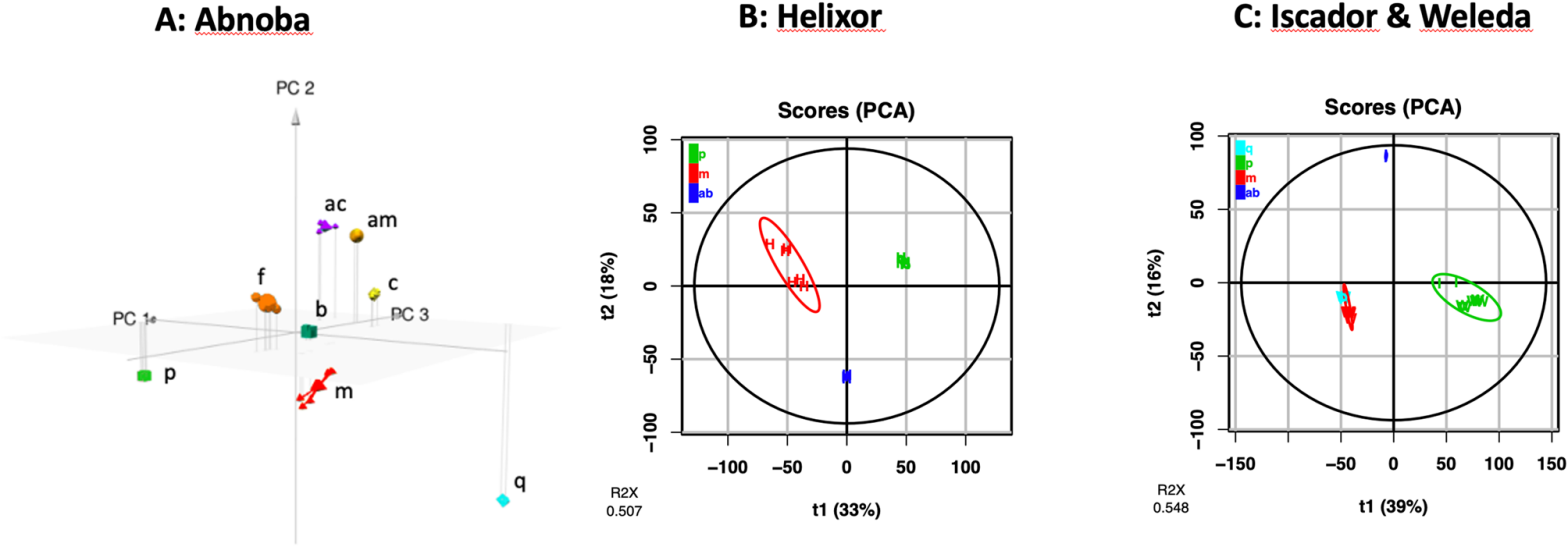
Principal Component Analysis (PCA) of *Viscum album* L. extracts based on LC–MS for each company: blue, *abietis* (ab); purple, *aceris* (ac); gold, *amygdali* (am); emerald, *betulae* (b); yellow, *crataegi* (c); orange, *fraxini* (f); red, *mali* (m); green, *pini* (p); cyan, *quercus* (q). (**A**) Abnoba products, 3D-PCA (PC1: 34%, PC2: 13%, PC3: 10%, R2X = 0.566), (**B**) Helixor products (PC1: 33%, PC2: 18%, R2X = 0.507), (**C**) Iscado & Weleda products (PC1: 39%, PC2: 16%, R2X = 0.548).

To better investigate the influence of the host tree on the composition of the extracts, the LC–MS data from each company samples were separately proceeded to consider if host trees have an influence on the metabolome (Fig. 3). Eight different Abnoba products (in triplicate) where 8 hosts are described were considered. With the same processing as described above, the 2 first-components of PCA model accounted for 47% of the variance in the sample set, the 3-first-components of PCA accounted for 56% of variance. The matrix obtained with this model was used to plot 3D-PCA as described in Fig. 3A. Indeed, a representation with the first three principal components allowed seeing the differences of the host within the same company.

Helixor products considered from 3 hosts and the pool of Weleda and Iscador products from 4 hosts were also analyzed. Their PCAs are described in Fig. 3B,C respectively. All PCAs have been obtained with the same treatment—Pareto scaling and log_10_ transformation. Here again, hosts influenced the composition of the manufactured products.

**Figure 4:**
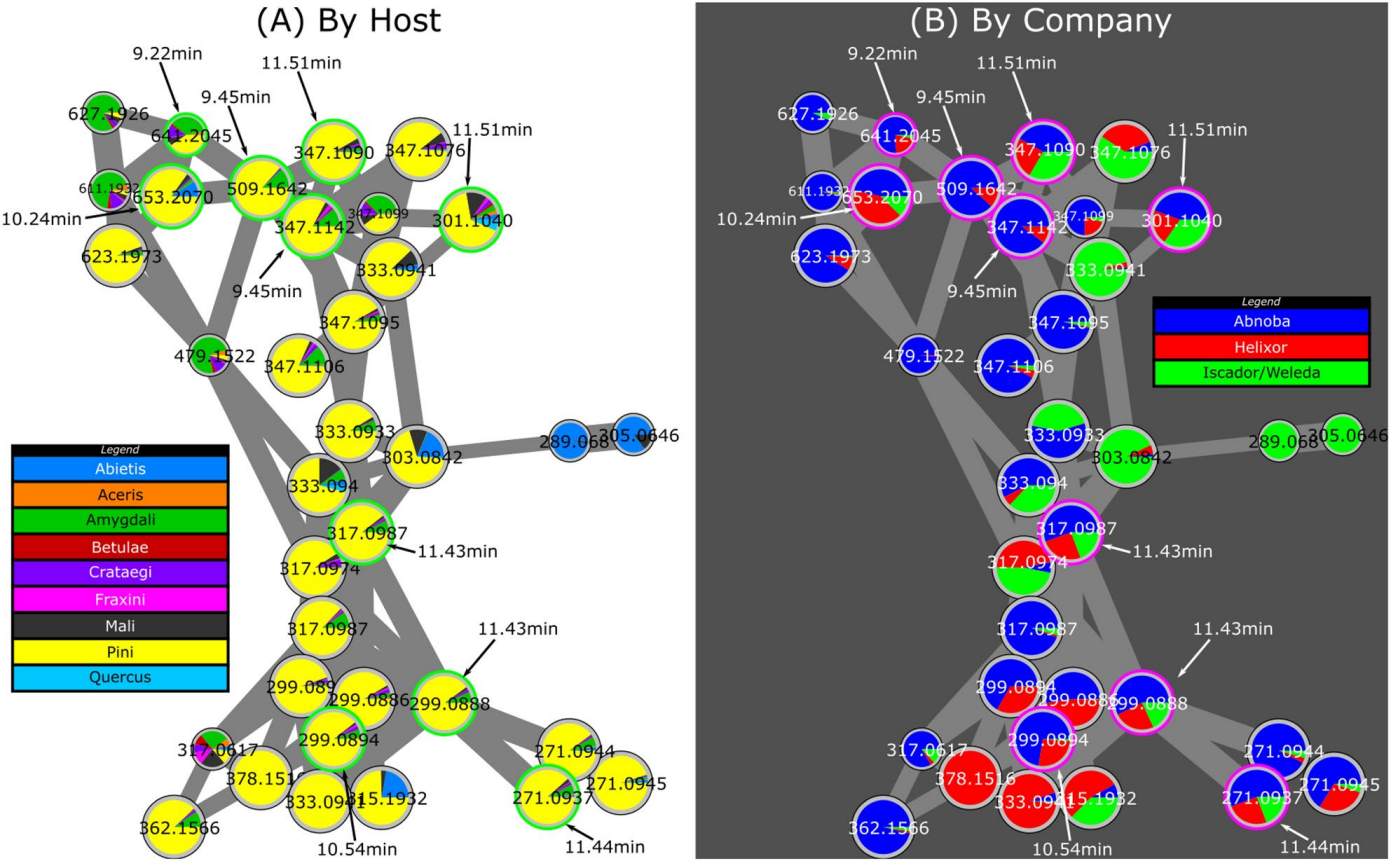
Molecular network of specialized metabolites annotated in crude extracts of *Viscum album* L. by LC–MS/MS analysis. Cluster related to isoflavonones (circled in green in Fig. 2). Pie charts are either related to the host (**A**) or to the producer (**B**). Green circles (left) and red circles (right) pointed with their respective retention time correspond to features discriminant for *pini* and common to two or three compagnies. Color legend for pie charts (**A**) and (**B**) are indicated on the side of the sub-cluster. See also Supplementary Table S2 for quantitative data.

As an illustration of the influence of the host, we were particularly interested in the *pini* host in relation to the biological results described below. From the molecular network results, the cluster related to isoflavonones (circled in green in Fig. 2) looks particularly interesting using another representation based on the host, with pie charts linked to the abundance of ions in crude extracts of *Viscum album* L. from different hosts. This cluster indicates that several nodes were mainly related to *pini* (Fig. 4A). In particular, 10 nodes pointed with their respective retention times were present in a cluster representative of *pini* and present in the different companies (Fig. 4, Supplementary Table S1). Using the standard search algorithm implemented in MetGem, several features could be annotated. Signal at *m/z* 271.0937 could be annotated as medicarpin with a cosine score of 0.65 from NIST14 database. Both signals at *m/z* 299.0888 and 299.0894 could be annotated as 2′-methoxyformonetin or 7-methylether retusin with a cosine score higher than 0.50 from MoNa database. Signals at *m/z* 317.0987 could be attributed to isoflavonones derivatives according to ISDB database (access code: UNPD 140103 and 214744) with cosine score higher than 0.45. Finally, isomers at *m/z* 347.1090 could be linked to isoflavonones using the analog search algorithm implemented in MetGem. Interestingly, focusing on isoflavonones, remarkable features helped to discriminate between producers. We notice that in this cluster we could identify markers of producers (in blue, green or red in Supplementary Table S2) at least in the batch studied (Fig. 4B). Fermentation was also detectable in this isoflavanone cluster since *m/z* 299.0894 rt 9.84 min or 347.1142, rt 9.45 min are present in Abnoba and Helixor but not in Iscador/Weleda *pini* products.

### Correlation metabolome and biological data

Vγ9Vδ2 T cells are unconventional T cells that possess several characteristics that make them attractive candidates for cancer immunotherapeutic approaches^30,31^. We have recently shown that mistletoe extracts can stimulate specifically this anti-cancer T cell subset^21^. Extracts studied were Ap, Am, Ip & Im and the Ap extract was the one that resulted in most robust activation of Vγ9Vδ2 T cells^21^.

Consequently, the chemical signatures or fingerprints of the four extracts were linked to their immunologic signature. We first analyzed features that were statistically more abundant in Ap by the Biosigner wrapper algorithm which classes molecular signature by 3 statistical models (with either the PLS-DA, Random Forest or SVM approach)^32,33^. Therefore, features (defined by their m/z and retention time values) that were present in at least two distinct Biosigner runs were selected.

**Figure 5:**
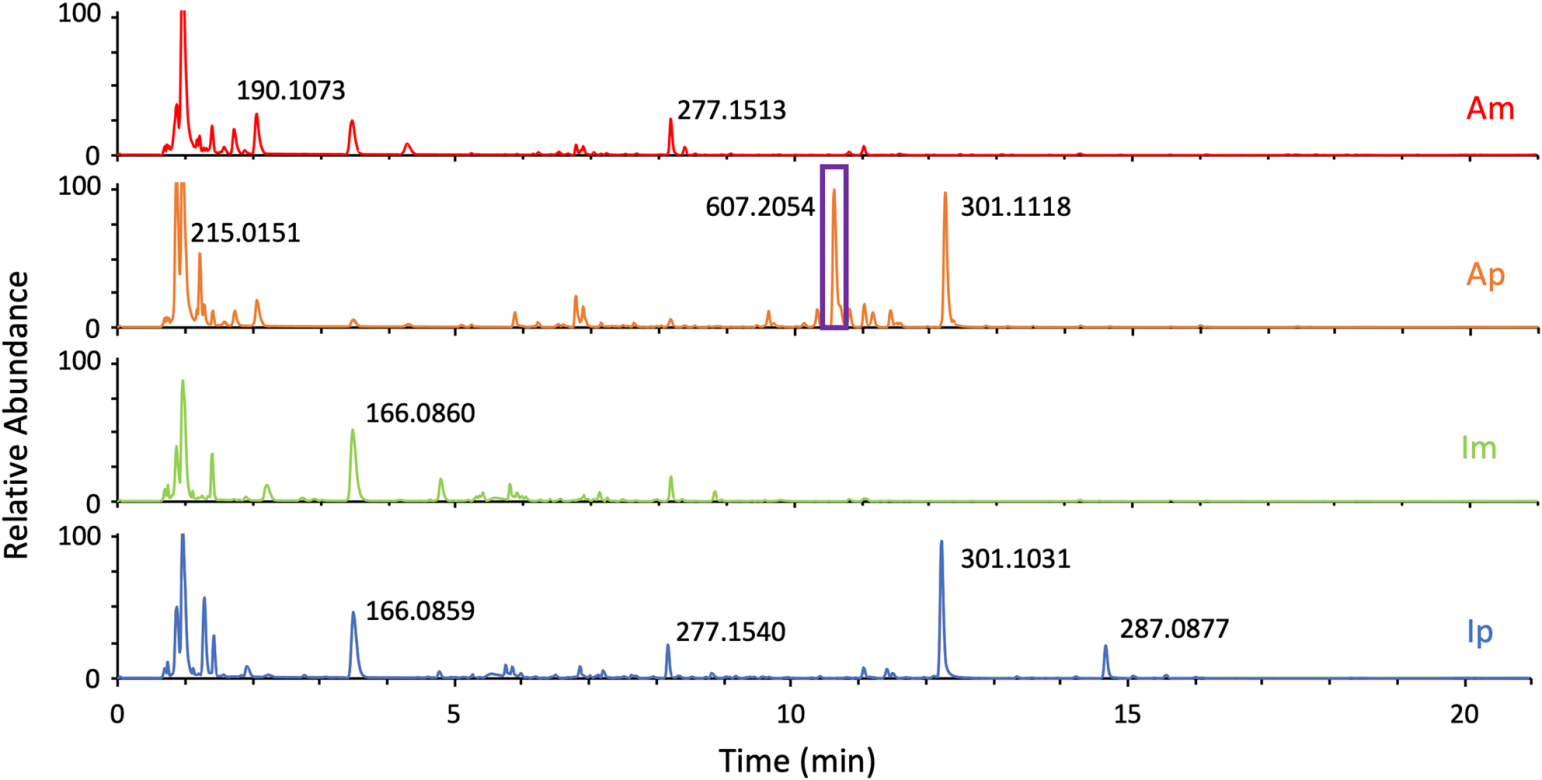
Comparison of TICs ESI + (Total Ion Chromatograms) of the selected extracts tested proliferation of Vγ9Vδ2 T cells. The feature identified by biosigner algorithm is framed on the Ap chromatogram.

A specific isotopic signature was particularly represented in Ap samples with *m/*z 607.2054 (δ ppm 5.38), rt 10.6 min (in positive mode) attributed to C_29_H_34_O_14_ molecular formula (Fig. 5). Conversely, the peak at 11.51 min m/z 301.1040 could be a marker of the *pini* host (Supplementary Table S2).

**Figure 6:**
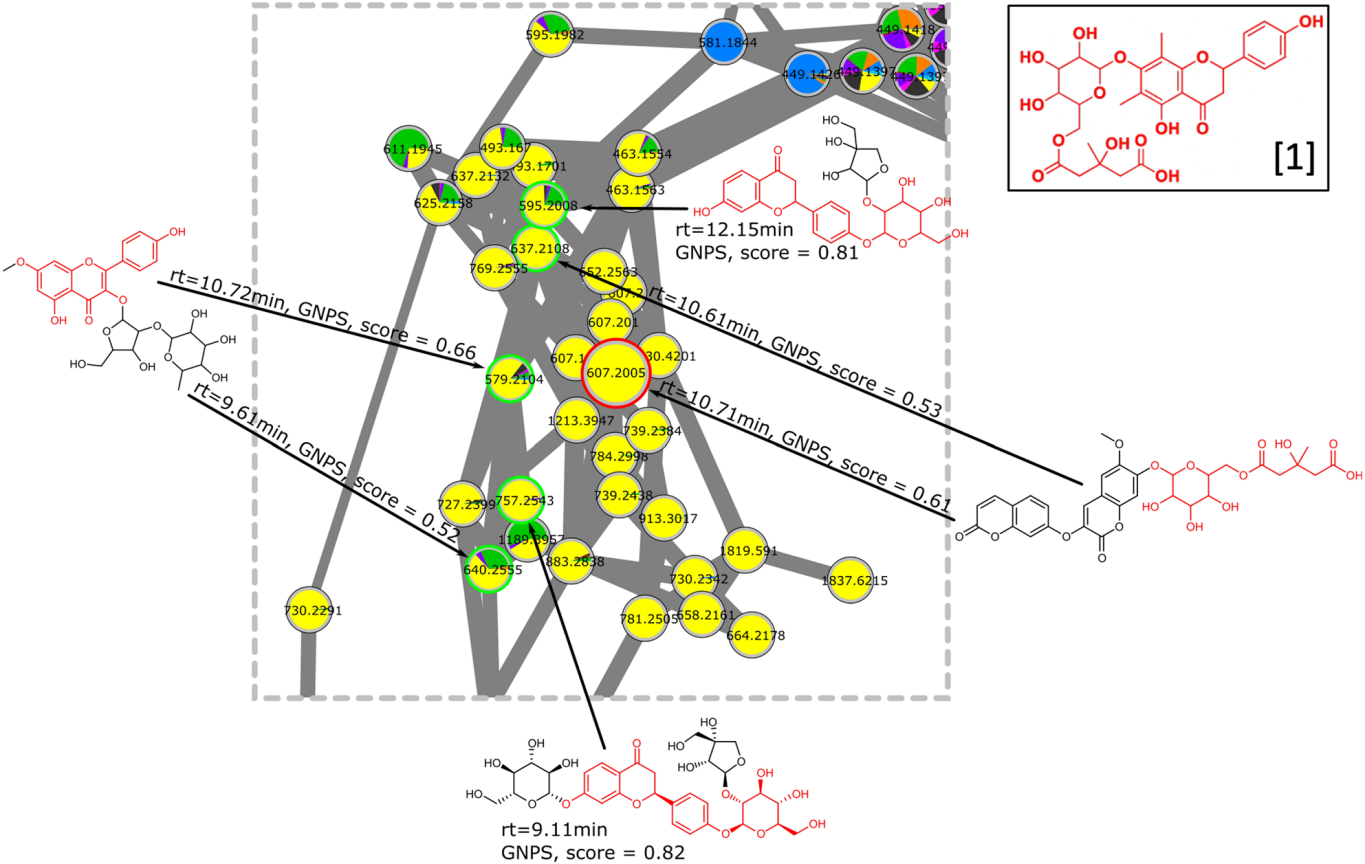
Molecular network of specialized metabolites annotated in crude extracts of *Viscum album* L. by LC–MS/MS analysis. Cluster related to glycosylated flavanones (circled in red in Fig. 2). Analogs (green circles) from database search implemented in MetGem software. Substructures colored red are in common with the structure of **[1]**, Supplementary Fig. S2 (node with the red circle). pie charts are related to the host (same legend as Fig. 4a).

Looking closer to the EIC allowed us to identify that the same *m/z* 607.2054 was also present at other retention times, which we interpreted as different isomers. Based on this observation, we decided to investigate by LC–MS/MS the chemical structure of this compound never described before in mistletoe. To provide a first annotation for the signal at *m/z* 607.2054 the cluster related to this particular ion (circled in red in Fig. 2) was carefully annotated using database search implemented in MetGem software. Six nodes were annotated as analogs of glycosylated flavanones with cosine score higher than 0.52 (Fig. 6).

In order to confirm this first annotation, targeted MS/MS experiments were carried out by selecting *m/z* 607.2021 (calculated value) as precursor ion in positive mode and m/z 605.1876 in negative mode. The exact mass and isotopic distribution in both modes suggested a structure C_29_H_34_O_14_ while major fragments were respectively *m/z* 301.1057 and 299.0917 in positive and negative mode with a suggested structure of C_17_H_16_O_5_ (**[1]**, Fig. 6 and Supplementary Fig. S2). First, the number of oxygens in the neutral lost during the fragmentation, suggested a glycosylated polyphenol. Second, the further fragmentations of the C_17_H_17_O_5_ structure were in accordance with a flavanone. Indeed, in negative mode, a fragment C_8_H_8_O (m/z 119.0500) corresponding to the 1,3B-fragmentation of the ring AC of a flavanone together with the corresponding fragment of ring AC in positive mode (C_9_H_8_O_4_, m/z 181.0479)^34^. Actually, this last fragment has been found in a dimethylflavanone compound according to Massaro et al. ^35^. In addition, the MS/MS network analysis operated with MetGem, localized in the same network this structure with compound wearing a 3-hydroxyl-3-methyl-glutaryl-6-O-glucopyranosyl branch (see above, Fig. 6). Looking further at structure of flavanone wearing this kind of branching, the 3-hydroxy-3-methyl-5-oxo-5-[[3,4,5-trihydroxy-6-[[5-hydroxy-2-(4-hydroxyphenyl)-6,8-dimethyl-4-oxo-2,3-dihydrochromen-7-yl]oxy]oxan-2-yl]methoxy]pentanoic acid (the phenolic analog of Matteuorienate A previously described)^36–39^. As a consequence, the fragmentation pattern of the structure in negative mode was compared with similar flavanone, namely melitidin and brutieridin, respectively described by Zou et al.^40^ and Di Donna et al.^41^ with a perfect match of the fragments.

To confirm this structure **[1]**, the Ap extract ^1^H-NMR spectrum has been carefully analyzed. Expected signals according to the literature for similar glycosylated flavanones have been looked forward in the complex matrix. The aromatic signals for 2′,6′ and 3′,5′ protons on the hydroxyphenyl were found at chemical shifts of 7.44 and 6.97 ppm respectively (Supplementary Fig. S3), which was very close of those reported for well-known Matteuorienate^36–39^. The ^3^*J*_H,H_ coupling constant observed for both doublets had the expected value of 8.9 Hz. The signals of both protons were related to the following buckets, B7.451, B7.431 and B6.951, for which more intense values are observed in Ap versus the other extracts studied. To identify the signals of both methyl protons, 6-CH_3_ and 8-CH_3_, in this spectrum, we relied on the NMR data matrix, the hierarchical clustering on NMR data (Supplementary Fig. S1A) and the spectrum of the extract. According to the spectrum (Supplementary Fig. S3), several singulet signals were present in the range 2.3 to 2.5 ppm that could correspond regarding to the intensity desired for both methyl signals. Two buckets, B2.448 and B2.408, were in the same branch of the hierarchical cluster than the three ones identified above (B7.451, B7.431 and B6.951). Indeed, there was every reason to believe that signals from a given molecule should be close to each other in the hierarchical clustering. These chemical shifts are quite different from those observed in the literature^36–39^ but this could be explained by the use of another analytic NMR solvent i.e., water here against acetone or methanol. The confirmation of the exact stereochemistry could be obtained on the purified product after LC-semi-preparative purification completed by 2D-NMR experiments.

The 4 extracts tested on Vγ9Vδ2 T cells were also analyzed by a PCA (Data not shown). Among the 15 most representative loadings in Abnobaviscum *pini* extract, we have found 2 buckets corresponding to the glycosylated flavanone **[1]**, B7.451 and B6.951. That confirms the importance of this molecule to discriminate Ap versus the other extracts.

Given the data obtained by NMR and MS, we hypothesized that the isomer **[1]** more abundant in Ap than in other mistletoe extracts is the one shown in Supplementary Fig. S2, in accordance with published structures described in the rhizomes of *Oriental Pentarhizidium*^38^ and *Matteuccia intermedia*^39^. Complete stereochemical identification cannot be made at this stage given the concentrations in the commercial extracts.

**Figure 7:**
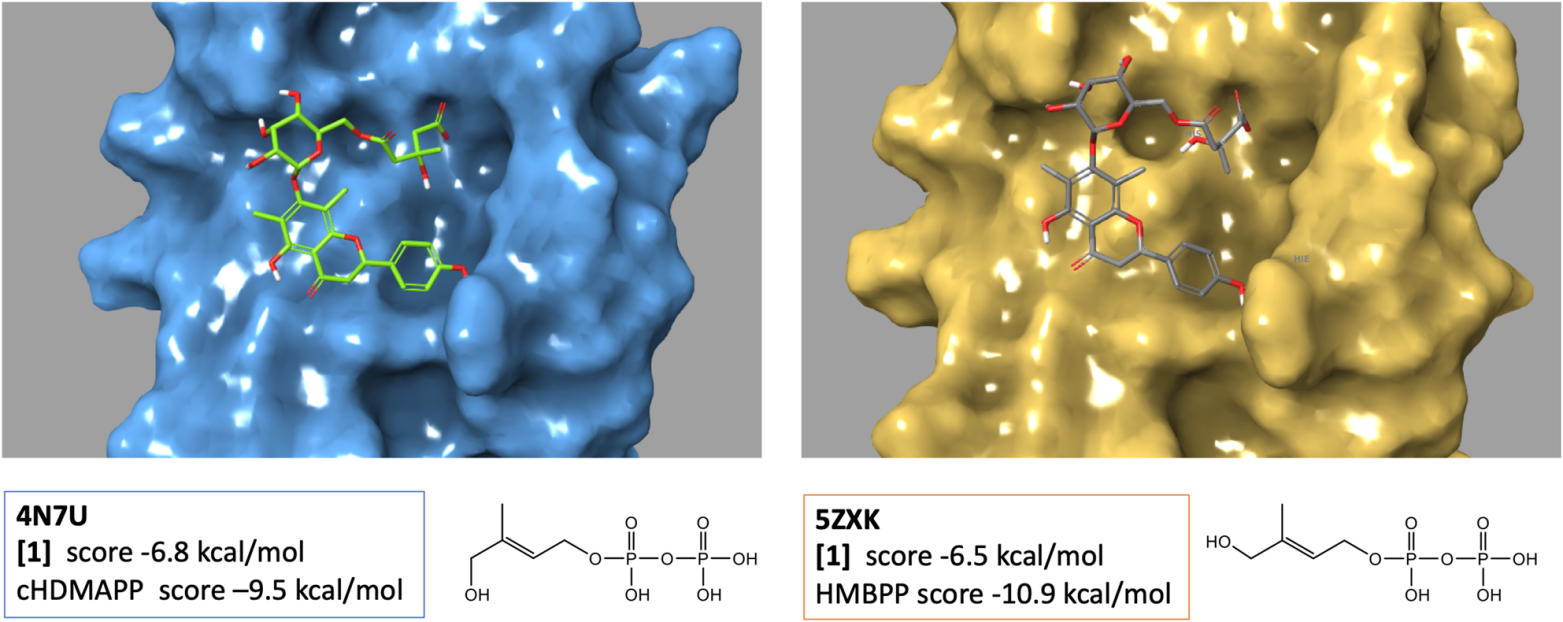
Molecular docking of compound **[1]** into the B30.2 domain of BTN3A1. (on the left) **[1]** binds to a basic binding pocket on the surface of the BTN3A1 B30.2 domain on published crystal structure (PBD ID 4N7U) (on the right) **[1]** binds to the crystal structure (PBD ID 5ZXK). Docking was performed using Schrodinger.

The activation of anti-cancer Vγ9Vδ2 T cells by Ap is dependent on the butyrophilin BTN3A1^21^ a transmembrane protein known to interact with phosphorylated metabolites (such as HMBPP, also known as phosphoantigens) via its intracellular domain B20.1^42^. It is thought that the conformational change induced in BTN3A1 by this intracellular interaction with metabolites is recognized by the Vγ9Vδ2 TCR thus resulting in the activation of Vγ9Vδ2 T cells^42^. Therefore, we wondered whether the glycosylated flavanone **[1]** can interact with the B20.1 domain of BTN3A1. We docked in silico **[1]** into two X-ray structures of this domain, in parallel with the native ligands (cHDMAPP and HMBPP) (Fig. 7). The best affinity score pose of each ligand obtained by docking in their respective Xray structure was very similar to their crystal position reflecting the capacity of our docking procedure to assess other compounds. The best score pose of **[1]** (Fig. 7) in both Xray structures formed similar interactions with the protein. The terminal carboxylate group of **[1]** which occupied the position of the terminal phosphate group of pAgs makes several salt bridges with three arginine residues (R412, R418 and R469) as previously described for the pyrophosphate group of HMBPP^43^. Hydroxyl groups of the sugar moiety hydrogen bond with Asp 407 and the hydroxyphenyl group of the flavanone forms a hydrogen bond with Trp350. The score of the **[1]** poses were also very similar in the two 3D structures: − 6.4 (in 5ZXK) and − 6.8 kcal/mol (in 4N7U) but lower than the score of the knowns phosphoantigens: cHDMAPP (-9.5 kcal/mol) and HMBPP (− 10.9 kcal/mol).

## Discussion

Metabolomics reveals the downstream products of proteins. The most frequently used tools are nuclear magnetic resonance (NMR) spectroscopy and mass spectrometry (MS) to perform metabolomics investigations. These high-throughput instruments play an extremely crucial role in discovery metabolomics to generate data needed for further analysis. In general, metabolomics technologies are undertaken to respond to questions in human disease studies, plant responses towards stress and abiotic resistance or applied to microbial metabolomics for biotechnology applications^44^. In this project, we have chosen to apply metabolomics technologies in one area of integrative oncology looking at CAM products used by patients and more specifically, commercial injectable preparations from the hemi-parasite plant *Viscum album* L. (European mistletoe). These commercial products are not drugs according to classic regulatory authorities (WHO, EMA…). Even if numerous cancer patients use this CAM in central European countries in search of therapeutic options^11,45^, the regulatory status of these products is unclear. If it is not a drug, it is logical for pharmacognosist experts to think that the composition is not standardized, but is this point clear for all medical professionals and patients?

To explore the metabolome of injectable mistletoe extracts preparation, we used complementary analytical tools that are routinely used, NMR and LC–MS, completed by LC–MS/MS for metabolite annotations. In addition, because of the complementary analytical features of NMR and MS, opportunities for leveraging both methods have been considered for more comprehensive metabolic profiling^46–48^. For this reason, all data have been processed by the same tool thanks to W4M platform^22^. It is clear that if the sensitivity of MS is incomparable with NMR and as a matter of facts the exact same metabolome was not observed, the visual inspection of the chemometric data via principal component analysis (PCA) revealed that both methods were able to group the commercial products according to their manufacturing process and host tree. It is interesting to notice how the PCA were similar whereas the nature and the number of variables were not comparable. Indeed, on one side the variables were 304 buckets for NMR and on the other side, the variables were 8928 pairs of retention times and m/z values. As a matter of fact, both analytical methods were able to differentiate products which allows analytical laboratories to choose their preferred analytical method for batch analysis.

Although the commercial extracts were claimed to be significantly different from one host tree to another, the most important factor of discrimination was the manufacturer with around 50% of the variance (Fig. 1). Thus, the major factor that provided a separation of the samples was the company. This could be explained by production process differences between companies. For example, Iscador and Weleda extracts have undergone a fermentation that could explain a metabolomic profile different from non-fermented Helixor and Abnoba. This observation is crucial for the general public. One may believe that while buying AbnobaVISCUM *mali* or AbnobaVISCUM *pini*, they will have quite different extracts whereas AbnobaVISCUM *pini* and Helixor *pini* will be closer. This is very confusing. Nevertheless, the host tree influence on the extract composition remains crucial from a scientific point of view but also for the anthroposophical professional. Here, both NMR (data not shown) and MS methods clearly separated the samples as a function of host into 8 very distinct groups by taking companies separately (Fig. 3). As far as other parameters are concerned, it is important to notice that manufacturers state that they mix preparations of mistletoe harvested at several times over the year (i.e. summer and winter) in order to have a homogenous production. As a consequence, environmental effects are averaged and only manufacturer processes and host tree effects were really observed as discriminant. Moreover, soil where host trees grew, climate, exact time of the harvest were not mentioned by the manufacturers but if they had influence on the chemical composition, we were unable to find such clusters with different batches in our statistical models.

To compare more precisely these groups related to the host tree, it is necessary to go further in the characterization of the variables (i.e., m/z and rt features) and access to molecular data. To annotate the variables, metabolomics community usually uses databases. In the case of *Viscum album,* databases are quite limited, so we looked to HRMS/MS and the resulting fragmentation data which can be organized as a molecular network. This approach aided in the recognition of structurally related chemical derivatives. Correlations were based on the MS/MS data of the respective metabolites on the principle that metabolites of similar chemical structures exhibit similar fragmentation patterns under identical ionization conditions^49^. This approach by fragmentation similarities is also suitable to decipher the molecular structure of metabolites of interest. For example, the MS/MS network was crucial to reveal the glycosylated flavanone **[1]** particularly abundant in AbnobaVISCUM *pini* (Ap) which was the mistletoe extract that showed the highest activation of Vγ9Vδ2 T cells in a BTN3A1-depentent manner^21^. In addition, this approach allowed us to identify families of parent metabolites (clusters) that were expressed according to a producer or a host tree. We identified a putative product **[1]** that was highly enriched in Ap compared to the other commercial mistletoe extract. It was characterized by LC–MS/MS and confirmed by ^1^H-NMR by identification of 5 pics in the spectrum (Supplementary Fig. S3). In literature, two products have been described where the hydroxyl group was described in 2′ or in 4′ position^38,39^. Considering ^1^H-NMR, we found that the hydroxyl group is in 4′ position.

Since Ap efficiently activates Vγ9Vδ2 T cells in a BTN3A1-depentent manner^21^, the 3D structure of **[1]** was docked into the phosphoantigen binding site of the intracellular domain structure of BTN3A1. The carboxylate group of **[1]** matched with the position of the terminal phosphate of the phosphoantigens^43,50^ as well as that of carboxylate of citrate and malonate^51^ observed in the crystal structures. In that position, the carboxylate group faced a triplet of arginines whose substitution was shown to be detrimental for binding^50^ and for HMBPP stimulation of Vγ9Vδ2 T cells. The docking score of **[1]** was however not as good as the scores of the phosphoantigens, cHDMAPP and HMBPP. This could be attributed to the ability of carboxylic acids to be mono-ionized unlike pyrophosphates, which provides more dipole–dipole/dipole–ion interactions. Further studies are needed to investigate directly the interaction of **[1]** with the B20.1 domain and/or the Vg9Vd2 T cell-activation capacity of **[1]** in order to confirm whether **[1]** is the main active substance responsible for the stimulation of anti-cancer Vγ9Vδ2 T cells by Ap.

## Conclusion

The mistletoe products are sold with the status of anthroposophic medicines in Germany and Switzerland to reinforce for example immunity in the context of cancer therapy. The EU regulatory picture for anthroposophic medicinal products is fragmented, and a very substantial number of these products are not yet part as such of the legislation in place in the European Union, nor in most of the Member States. In fact, as shown in this study, mistletoe products do not meet the standard criteria for drug legislation as the chemical composition varied from a producer to another. Indeed, the main determinant in terms of chemical composition was the manufacturer before the host tree. This information does not seem really clear to clinicians and literature reviews that generally consider equivalent all mistletoe products from all manufacturers in a specific therapeutic frame. As far, scientific results are unable to form an objective view of these products. Yet some of these products selectively have promising biological activities. It therefore seems necessary to move towards more standardized products and more rigor regarding these plant-based CAM and elucidating the compound(s) or *totum* that are responsible for the biological and clinical activities.

## Methods

### Sample and QC preparation for LC–MS and NMR experiments

Abnoba, Helixor and Iscador products have been supplied free of charge by companies. Weleda France did not answer, and all Weleda products were purchased in a French pharmacy. The batch numbers were respectively 708G01, 801F23, 712D18, 712F16, 710A08, 711C15, 801A20 for Aac, Aam, Ab, Ac, Af, Am, Ap, and Aq, 4170702, 4170805, 4170606 for Hab, Hm, and Hp, 5341/02, 6094/02, 6206/02 for Iab, Im, and Ip, 000628F0, 006331F0, 006317F0 for Wm, Wp, and Wq. The samples information is summarized in supplementary Table S1.

For NMR experiments, each extract was prepared at a concentration of 9 mg/mL in H_2_O/D_2_O 90/10 with TSP-*d4* at 0.25 mM for chemical shift calibration. A QC sample was prepared by mixing equal volumes (25 µL for extract at 10 mg/mL) from each sample to validate the stability of NMR analysis.

For LC–MS experiments, the samples at 50, 20 or 10 mg/mL were diluted in aqueous formic acid 0.1% (v/v) to obtain solutions at 10 mg/mL. A quality control (QC) sample was prepared by mixing equal volumes (40 µL) from each sample to validate the stability of LC–MS sequence analysis.

### Nuclear magnetic resonance

NMR data were obtained at 500 MHz on a Bruker Avance III NMR spectrometer equipped with a Prodigy CryoProbe from the Plateforme Chimie Nanobio of the Institut de Chimie Moléculaire de Grenoble (FR2607). ^1^H experiments were performed at 25 °C with the sculpting scheme for water suppression using a selective pulse of 1.9 ms. The acquisitions were performed over a spectral width of 15 ppm, with a FID size of 32k points and 384 scans/experiments. All fid data (17 samples with duplicates for 6 of them) were acquired the same day.

Preprocessing of the data (phase correction, solvent suppression, apodization, Fourier transform, shift referencing, baseline correction), spectra alignment, bucketing, normalization, quality control (metabolites correlation analysis), and statistical analyses (univariate testing and multivariate modeling) were conducted on the online and freely available Workflow4Metabolomics (W4M platform; https://workflow4metabolomics.usegalaxy.fr/). For extracts with two replicates, the data matrix was built with the mean of duplicates. Spectra alignment was performed only for the following spectrum areas: 3.6344–3.1041 ppm, 2.1048–2.004 ppm, 1.3949–1.0047 ppm. The solvent spectrum region was excluded (from 5.1 to 4.65 ppm). The bucketing was applied with a bucket width of 0.02 ppm on a spectrum region from 8.7 to 0.85 ppm. After the normalization step, no significant buckets (peak area inferior to 0.0003) were excluded from the data matrix, which are: B8.69/B8.671/B8.63 to B8.511/ B8.431/B8.411/B8.35/B8.291 to B8.23/B8.171 to B7.911/B7.851/B7.791/B7.73/B7.29/B7.03/B6.831/B6.751 to B6.691/B6.57/B6.55/B6.511/B6.151 to B6.07/B6.03/B5.89/B5.871/B5.71 to B5.65/B5.491/B5.371/B5.31/B5.21/ B5.171 to B5.111/B3.173/B2.952.

### Liquid chromatography

Chromatographic analysis was performed using a UPLC Vanquish Flex Binary system (Thermo Fisher Scientific, Waltham, MA, USA). The column used for chromatographic separation was an Accucore RP-MS column (2.1 mm × 150 mm, 2.6 μm, Thermo Fisher Scientific, Waltham, MA, USA). The mobile phase was constituted of aqueous formic acid 0.1% as phase A and acetonitrile formic acid 0.1% as phase B using gradient elution at a flow rate of 0.4 mL/min. The following gradient was applied: 0–2 min, 1% B; 2–20 min, 1–75% B; 20–20.1 min, 75–100% B; 20.1–22 min, 100% B; 22–22.1 min, 100–1% B and 22–26 min, 1% B. The injection volume was 10 μL and the column temperature was maintained at 30 °C. All samples were injected in triplicate in a randomized order to avoid deviations caused by injection order. The QC sample was injected at the beginning and the end as well as along the analytical run.

### Mass spectrometry

Mass spectrometric measurements were achieved by a Q-Exactive Plus Orbitrap MS (Thermo Fisher, Waltham, MA, USA) using a heated electrospray ionization source in the positive ionization mode controlled by Excalibur software. Calibration was performed before analysis using a positive calibration mix (Piercenet—Thermo Fisher, Rockford, IL, USA). Tune parameters were as follows: spray voltage, 5 kV; sheath gas flow rate 60 (arbitrary units); auxiliary gas flow rate 10 (arbitrary units), capillary temperature 280 °C, auxiliary gas heater temperature 400 °C. The metabolomics data were obtained with the MS detector in full-scan mode (Full-MS) and with data-dependent acquisition (dd-MS2) for the top-8 most abundant ions per scan and stepped normalized collision energies of 35 eV. Full MS and dd-MS2 spectra were recorded from m/z 100–2000 with a resolution of 70,000 and 17,500 (at *m/z* 200), respectively.

### Structure elucidation

MS/MS: The elucidation of the structure was performed on an Agilent QTOF 6520 coupled to a LC1200 (Agilent technologies, Santa Clara, CA, USA). Briefly, the compounds were separated with a Poroshell 120, EC-C18, 2.1 × 100 mm, 2.7 μm column, preceded by a Poroshell 120, EC-C18, 2.1 × 5 mm, 2.7 μm guard column, using formic acid 0.1% in water/acetonitrile with 0.1% of formic acid gradient (1% acetonitrile for 2 min increasing to 75% in 18 min and to 100% in 2 min, then kept at 100% for 4 min. Before decreasing to 1% for 0.2 min, the column was reequilibrated for 3 min). The TargetMS/MS mode was operated with the following parameters: source; gas temperature 325 °C, Drying gas 9 L/min, nebulizer, 45 psig, VCap 4500 V, fragmentor 175 V. The precursor ions selected were respectively m/z 607.2027 and m/z 605.1876 for positive and negative mode with an isolation window of ± 4 amu. The collision energies of 5, 15, 25, 40 and 55 eV were applied during the run and MS/MS range spectra were between m/z 50 and 1000. The data were analyzed with MassHunter qualitative analysis version B.07 from Agilent Technologies where precursor and product ions structures were estimated.

### Data processing and statistical analysis for LC–MS

Thermo Fisher .d format data were converted to .mzXML format using the ProteoWizard MSConvert tools (Version 3.0.19057-5e3190638, 64-bit) with the Peak Picking filter option. Preprocessing of the data (automatic peak detection, integration, peak filtration, peak identification, peak grouping and smoothing, retention time correction, integration, annotation), normalization (batch correction), quality control (metabolites correlation analysis and determination of batch correction), and statistical analyses (univariate testing and multivariate modeling) were conducted on the online and freely available W4M platform. Briefly, preprocessing was performed by using the implementation of the XCMS software^52^ in W4M. The “centWave” algorithm^53^ was used with the parameters adapted for an Thermo LC-QExactive. Intensity drift correction was performed using a local quadratic (loess) model that represents the intensity variation along injection order using the QC sample^54^. Variables were then filtered to remove those with a mean intensity that was lower than twice the mean intensity in reagent blanks, or variables with a coefficient of variation in the QC samples above 30%. Principal component analysis (PCA) was used for multivariate exploration of clusters and trends among the observations.

Supervised partial least-squares discriminant analysis (PLS-DA) models were also built (the significance of the Q2Y prediction performance metric was assessed by comparison with 20 models built after random permutation of the response values). Hierarchical clustering of samples and variables (heatmap) was performed by using the 1-cor dissimilarity (where cor is the Spearman correlation) and the Ward’s linkage method. The variables that are significant for the classification performances between commercial origins (with either the PLS-DA, Random Forest or SVM approach) were selected with the Biosigner wrapper algorithm^32^. Since Biosigner relies on an internal resampling approach, re-running of the module may result in slightly distinct signatures. Therefore, features (defined by their *m/z* and retention time values) that were present in at least two distinct Biosigner runs were selected. All statistical analyses were performed on the W4M infrastructure, unless otherwise specified.

### Molecular networking processing

#### MZmine 2 data preprocessing parameters

Networks were generated using the methodology previously described by Olivon et al.^23^. Mass detection was realized keeping the noise level at 3.0E5 for MS level 1 and 0 for MS level 2. ADAP chromatogram builder was employed using a minimum group size of scans of 5, a group intensity threshold of 3.0 × 10^5^, and an m/z tolerance of 0.01. ADAP wavelet deconvolution algorithm was used with the following standard settings: S/N threshold of 10, minimum feature height of 3.0 × 10^5^, coefficient/area threshold of 10, peak duration range of 0.05–0.6 min, and RT wavelet range of 0.01–0.15. MS2 scans were paired using a m/z tolerance range of 0.01 Da and a RT tolerance range of 0.1 min. Isotopologues were grouped using the isotopic peak grouper algorithm [m/z tolerance of 20 ppm, RT tolerance of 0.1 min, maximum charge of 3]. For peak alignment, step was performed using Join aligner module [m/z tolerance of 20 ppm, weight for m/z of 75, RT tolerance of 0.1 min, weight for RT of 25]. The resulting peak list was filtered to exclude the first 70 s (to remove molecules not retained in the column) and then gap-filled with the “same RT and m/z range gap filler” module [m/z tolerance of 20 ppm].

Eventually, .mgf preclustered spectral data files and their corresponding .csv metadata files (for RT, areas, and formula integration) were exported using the dedicated “Export for GNPS” and “Export to CSV file” built-in options.

### MetGem parameters

Cosine score and molecular networks were calculated on MetGem 1.3.4 software^24^. MS/MS spectra were window-filtered by choosing only the top 6 peaks in the ± 50 Da window throughout the spectrum. The data was filtered by removing all peaks in the ± 17 Da range around the precursor *m/z*. The *m/z* tolerance windows used to find the matching peaks was set to 0.02 Da and cosine scores were kept in consideration for spectra sharing 2 matching peaks at least. The minimal cosine score value to link two nodes was fixed at 0.65.

For standard search in databases (GNPS^25^, MS-Dial^26^ and ISDB^27^), parameters were as follows: *m/z* tolerance = 0.2 Th; minimum matched peaks = 2; minimum intensity = 0%; *m/z* parent tolerance = 17 Th; min. cosine score = 0.45. For analog search, the same parameters were used and *m/z* tolerance for the precursor ion was set at 100.

### Docking protocols

The X-ray structure of human intracellular domain (B30.2) of BTN3A1 determined in complex with HMBPP (PDB ID: 5ZXK)^43^ and with docked cHDMAPP (PDB ID: 4N7U)^50^ were used as the target structure to endeavor the docking studies. The X-ray water and other ligand molecules were removed from the active site. The protein structure was prepared using the Protein Preparation Wizard (Schrödinger Release 2017-1: Protein Preparation Wizard; Epik, Schrödinger, LLC, New York, NY, 2020; Impact, Schrödinger; Prime, Schrödinger). The ligand input files were prepared according to the following procedure. The initial 3D structures of the ligands were generated using the Ligprep module from Schrodinger (Schrödinger Release 2017-1: LigPrep, Schrödinger). The Epik program was used to predict the different protonation states of all ligands (Schrödinger Release 2017-1: Epik, Schrödinger). Docking was performed using the Glide XP docking protocol and scoring function which approximates a systematic search of positions, orientations, and conformations of the ligand in the receptor binding site using a series of hierarchical filters (Schrödinger Release 2017-1: Glide)^55^. The binding region was defined by a 22 Å × 22 Å × 22 Å box centered on the central position either of CHDMAPP or HMBPP ligand in the crystal structure. At most 10 poses were generated for each molecule. All otter default settings were used.

## Supplementary Information

Supplementary Information.
