# Supplementary material for: Untargeted metabolomics approach to discriminate mistletoe commercial products
Source: Sci Rep. 2021 Jul 9;11:14205. doi: 10.1038/s41598-021-93255-z (PMC8270909; doi:10.1038/s41598-021-93255-z)
Supplement: Supplementary file 1 — Supplementary Information. [file 41598_2021_93255_MOESM1_ESM.pdf]

Title: Untargeted metabolomics approach to discriminate mistletoe commercial products

Author names: Cécile Vanhaverbeke, David Touboul, Nicolas Elie, Martine Prévost, Cécile Meunier, Sylvie Michelland, Valérie Cunin, Ling Ma, David Vermijlen, Cédric Delporte, Stéphanie Pochet, Audrey Le Gouellec, Michel Sève, Pierre Van Antwerpen and Florence Souard

Brands	Hosts	Abreviation	**Concentration**	**Lot**	Replicates
**Helixor**	*Abietis*	ab	50 mg/l	4170702	3
	Mali	m	50 mg/l	4170805	3
	Pini	p	50 mg/l	4170606	3
**Abnoba**	*Abietis*	ab	20 mg/l	710E09	3
	Aceris	ac	20 mg/l	708G01	3
	Amygdali	am	20 mg/l	801F23	3
	Betulae	b	20 mg/l	712D18	3
	Crataegi	c	20 mg/l	712F16	3
	Fraxini	f	20 mg/l	710A08	3
	Mali	m	20 mg/l	709A06<br>605A47	3<br>3
	Pini	p	20 mg/l	711C15	3
	Quercus	q	20 mg/l	801A20	3
**Iscador**	*Abietis*	ab	20 mg/l	5341/02	3
	Mali	m	20 mg/l	6094/02	3
	Pini	p	20 mg/l	6206/02	3
**Weleda**	*Mali*	m	20 mg/l	000628F0	3
	Pini	p	20 mg/l	006331F0	3
	Quercus	q	20 mg/l	006317F0	3

Supplementary Table S1: Details of the different batches used in the study. The concentration is referred to fresh plant material. According to manufacturers, mistletoe has been collected in summer and winter on the different host trees. Manufacturers precise that both extracts were prepared separately before mixing them with the view to obtain a constant production. It is noteworthy that Weleda marketed Iscador products meaning that two batches were analyzed for Iscador *mali* and *pini*.

https://www.iscador.com/en-de/mistletoe-preparations/production.html
https://www.abnoba.de/wp-content/uploads/2019/07/Pat_Info_web_E_2014-1.pdf
https://www.helixor.com/integrative-cancer-therapy/mistletoe-therapy/manufacturing/

Features		Abnoba (%)	Helixor (%)	Iscador/ Weleda (%)
m/z	rt (min)			
**271.0937**	**11.44**	**56**	**25**	**19**
271.0944	10.45	93	3	4
271.0945	9.83	66	29	5
299.0886	10.59	52	47	1
**299.0888**	**11.43**	**57**	**25**	**18**
299.0894	9.84	67	33	0
**299.0894**	**10.54**	**72**	**26**	**1**
**301.1040**	**11.51**	**44**	**21**	**35**
303.0842	9.09	2	5	93
315.1932	11.23	8	54	38
317.0974	11.43	6	47	47
317.0987	10.47	95	1	4
**317.0987**	**11.43**	**55**	**25**	**20**
333.0933	10.43	58	0	42
333.0940	10.14	57	6	37
333.0941	11.17	5	94	1
333.0941	9.22	1	3	96
**347.1090**	**11.51**	**42**	**25**	**33**
347.1095	10.47	96	1	3
347.1099	9.22	75	21	4
347.1106	8.31	93	4	3
**347.1142**	**9.45**	**90**	**10**	**0**
362.1566	10.45	96	1	3
378.1516	11.17	0	100	0
**509.1642**	**9.45**	**88**	**12**	**0**
623.1973	10.17	91	8	1
**641.2045**	**9.22**	**76**	**21**	**3**
**653.2070**	**10.24**	**47**	**41**	**12**

Supplementary Table S2 Based on LC-MS/MS data 28 representative more abundant features or markers in mistletoe extracts with *pini* host. The features in bold correspond to the 10 that are discriminant for *pini* and common to two and three compagnies, and pointed with their respective retention times in **Fig. 4**.

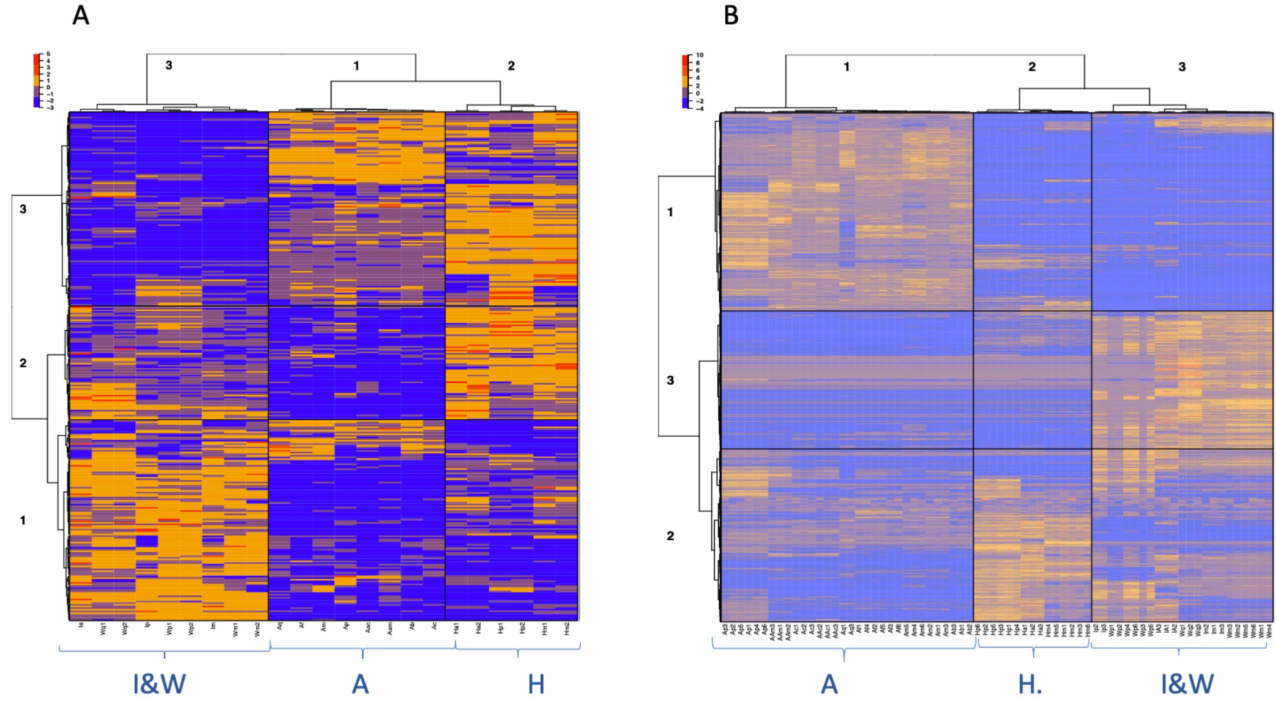

Supplementary Figure S1. Heatmap visualizations constructed based on the differential metabolites of importance for mistletoe extracts. Heatmap represents unsupervised hierarchical clustering of samples (columns) and variables (rows), on the left based (A) on H^1-NMR buckets, on the right based (B) on LC-MS features.

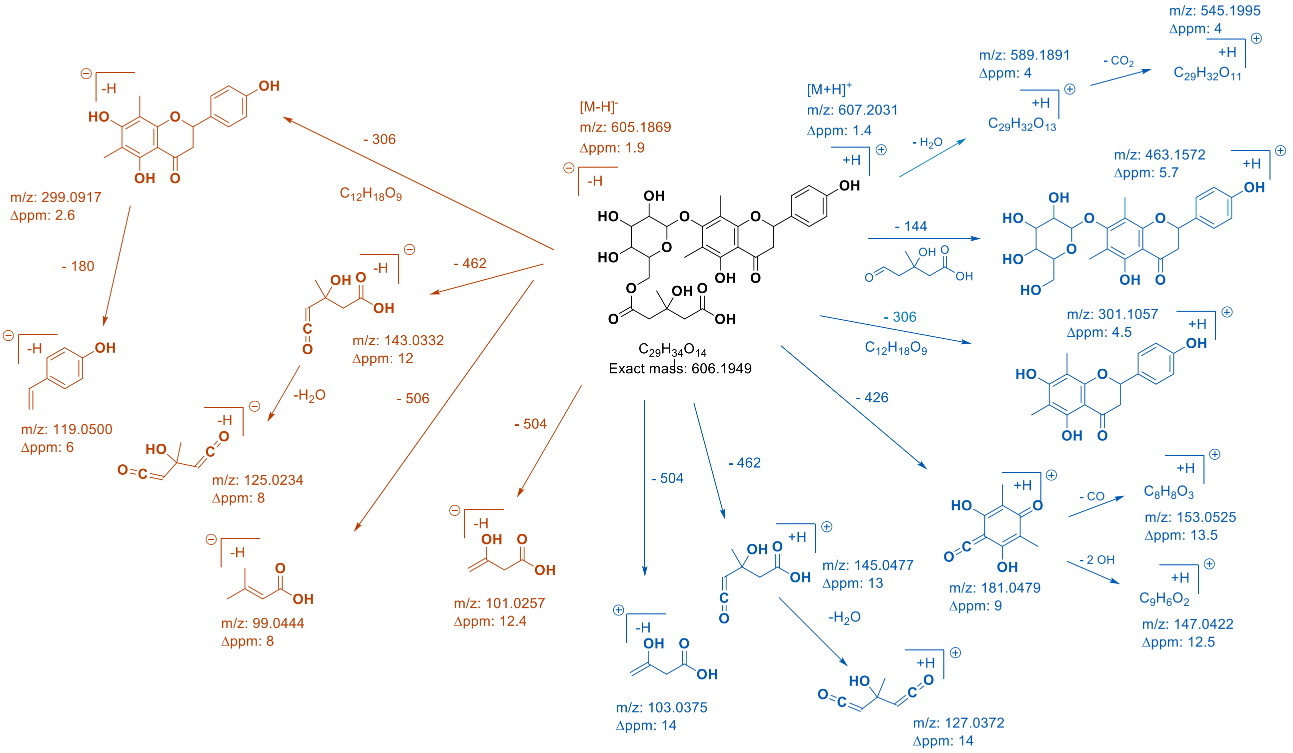

Supplementary Figure S2. MS/MS fragmentation pathways of the glycosylated flavanone **[1]** (in black) identified in Ap extracts. Negative mode is in red and positive mode in blue.

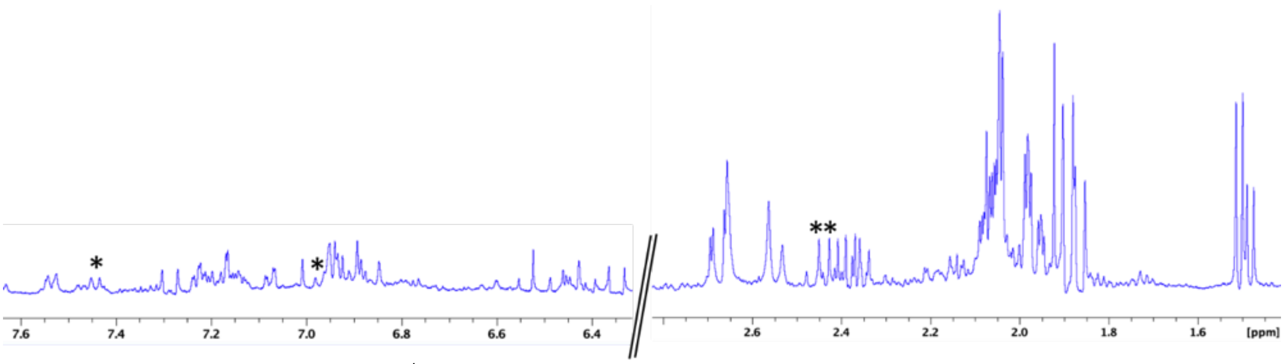

Supplementary Figure S3. 500 MHz ^{1}H-NMR spectrum of Ap extract at 25 °C. Only the 1.4 to 2.8 ppm and 6.5 to 7.6 ppm regions are represented. Putative signals of the glycosylated flavanone **[1]** are marked with a star.
